# Supporting and leveraging science as a social activity: The value of sharing ideas early and often

**DOI:** 10.1007/s40037-021-00653-0

**Published:** 2021-02-11

**Authors:** Glenn Regehr

**Affiliations:** Centre for Health Education Scholarship, Faculty of Medicine, University of British Columbia, Vancouver, Canada

In the Insider’s Perspective section, an insider in health professions education offers his/her thoughts, contemplations and advice on readers’ dilemmas or questions. You can send your questions or dilemmas to lieda.meester@bsl.nl. And who knows, your question may be the topic of the next instalment of the Insider’s Perspective.

*Dear insider, my research supervisor has suggested I contact a PhD student from another university who studies a topic similar to mine to discuss ideas. I fear that sharing my ideas and plans could mean she publishes her work before I do. She might even use my ideas for her plans! Can you advise me on this?*

When you are developing and nurturing a particularly fine idea, it can feel precious and hard won. Moreover, many of us have heard stories of academic communities where one would never risk sharing an idea for fear that it might be stolen before you are ready to share it publicly or shredded before you are ready to defend it properly. And many of us, even now, dwell in academic institutions that spout a rhetoric of collaboration but continue to reward individual success and recognition. So, it is understandable that you might have an instinct to protect your idea until you are fully satisfied with it yourself, to hide it until you are ready to reveal it to the world in its perfected form through a formal publication or presentation. However, I would argue that this is neither the best way to develop ideas, nor the best way to spread them.

In the global community of health professions education, there is a strong culture of collaboration and generativity. There is also a spirit of supporting others to grow and evolve their ideas (and their careers). And there is a general belief that the best way to move the field forward is through collective effort rather than individual competition. As a result, ideas are often shared back and forth quite freely in casual conversation. This is not to say that we are the perfect utopia. So, seeking your supervisor’s (or mentor’s) advice about when, where, and with whom you might first share an emerging idea makes a lot of sense. But, even if it might feel risky, the potential benefits of sharing your ideas early and often will outweigh the potential concerns.

Perhaps the most obvious and immediate personal benefit of sharing your early ideas is that those ideas, and your ways of presenting them, will be better for it. The formal peer review process in any scholarly domain is slow and spotty at best. By contrast, the peer review you get from sharing your idea in a conversation is immediate and often rich in information. You can see where a person’s eyes light up, where they frown, and where their eyes glaze over. Thus, you can quickly discover what wording and framing of the problem creates understanding or confusion, engagement or boredom, and relevant connections or misinterpretations. Conversations allow you to continue negotiating your position in real time. You can try different approaches to make your case, clarify your language, nuance your description, and manage or accommodate concerns that people raise. Beyond merely honing your language, these conversations will inevitably improve your ideas and generate new insights that you might never have imagined. Even just trying to express your thoughts out loud and hearing others’ perspectives on the issue can enrich your thinking. As an added bonus, if you properly engage the person, you are likely to get powerful, real life examples that you can incorporate into your future discussions. And you may well hear about relevant literature and authors that you would not have otherwise considered or explored. Thus, you are likely to come to a more nuanced and more elaborated version of your idea than you had before the conversation.

Conferences, by the way, are a great place to accomplish this. If you are conferencing well, you will connect (or reconnect) with many people in hallways and informal meeting spaces. Each of these moments is a chance to test drive an idea. When they ask you “What’s new?” or “What have you been up to?” you can answer with, “Well, I have been playing with this idea about …” and try out your one-minute pitch of the idea (what some have called the elevator pitch since you should be able to make your case as you ride up an elevator knowing your audience may get off at any time). If the response is something like, “Interesting … and have you had a chance to see any of the sights of Glasgow yet?” then you can have some confidence that you don’t have the pitch right. But if they lean in and say, “Huh, that reminds me of a paper I read the other day …” or “Oh, that sounds like something that happened to me once …” then you are on track and get to see where the discussion leads. Over the course of a three- or four-day conference, you can have several such conversations about the idea, updating your language, your examples, and your idea each time. Thus, you can rapidly develop a framing that engages people, a variety of examples to compellingly concretize the issue, and an array of leads for other authors you should track down. Through these iterative refinements, not only will your understanding of the idea be better, you will also be much better prepared to write a compelling introduction to a paper, one that will engage the audience you seek to engage by framing the problem in a way that you already know will intrigue them.

Another benefit of sharing early and often is the extent to which you can help people understand how your idea might fit with the work that they are doing. In this way, you are “preparing the ground”, increasing the chances that your ideas will land in fertile soil when you publish. Thus, what might be considered a potential concern (that others adopt your ideas without due credit) can be seen instead as an opportunity (preparing people to take your ideas up when *you *publish). As an example of this, I was speaking with a colleague who has recently entered the field of health professions education from another discipline and is trying to articulate her ideas in a way that is relevant to our community. She had shared her ideas with another senior colleague who is well connected to a national medical education organization. A few weeks later, she heard a leader from that organization promoting her ideas as an important direction in which the community must move but attributing the ideas to the senior colleague. The young researcher was deeply concerned that her ideas had been appropriated. But in our conversation, she came to realize two things. First, she need not assume that the senior colleague “stole” her idea. It may well be that the senior colleague did mention her name in the conversation with the leader, but that the leader simply shortened the chain of attribution in his casual statements. Assuming so, the senior colleague will likely ensure that, when it matters, credit for the idea will be directed to where it belongs. This may not be the case, but the point is to not assume the worst of the senior colleague before you know more about the situation. Second, she came to understand that this leader in medical education had just put out a call for the very paper she was in the process of writing, and she was uniquely positioned to move this agenda forward (it was, after all, her agenda). So, she became even more excited to get her “manifesto” out for publication, thereby answering the call that she, herself, had (unintentionally) initiated. In essence, she was able to ride the wave of enthusiasm that she had previously generated through her conversation with key players in the field.

Taking the idea of “preparing the ground” even further, you can be quite intentional about this process in your conversations. If you have had meaningful conversations about the work with others and helped them understand how your work informs theirs, then once you publish, your paper becomes highly citable. People will remember your conversation (and you can remind them of it by sending them the paper once it is published), and they will recognize your work as a good citation for a point they are trying to make. Thus, rather than simply tossing an idea into the literature and hoping it gets recognized and taken up, you can be proactively preparing people for the idea in advance: informing them not only that it is coming, but how to use it in their own work and publications.

Of course, there is always a possibility that, through conversations of this sort, someone could (either intentionally or inadvertently) incorporate your ideas into their own work and “scoop” you on the ideas. However, this would seem to be a very low risk. I can think of only two instances in my own past where a student came to me with such concerns, but neither seemed justified on closer inspection. One appeared to be an example of coincidental convergence on a pithy phrase, but the term was being used with a very different meaning in the other author’s work. The other was likely an over-interpretation of the similarity of ideas, and on closer inspection the other author had not captured the nuance of the student’s work. In both cases, the students’ publications have been taken up well in the literature, and the incident seemed not to have affected this success. But even if the other person’s use of your ideas does overlap with your own, it is not clear that this is a serious problem. As with the second case above, you likely have a more nuanced version of the issue because of the conceptual work you have put in. So, again, it might be best to think of this situation as the other person “preparing the ground” for your work. Most likely you will be able take the work forward with greater ease and publicity than if you were the only one writing about it. It is true that some credit may go to the person who “said it first”, but credit will also accrue to the person who leverages the ideas well in the long term, and you are in the best position to leverage your ideas well.

Finally, being free in sharing your early thinking with others can create a reputation for being open and generous with ideas, which has its own value. It enables others to be more free with you about their ideas, further enriching your own thinking as well as your awareness of what is going on in the field. It creates greater opportunities for collaboration as you find others with related interests. And it positions you as a contributor to (rather than detractor from) the very culture of collaboration and generativity that makes this community such a special place to spend time. Science, at its best, is a social activity. Social activities, at their best, involve meaningful personal engagements. And meaningful personal engagements are open, friendly, and mutually supportive. In health professions education, there is a culture of friendliness and mutual support. You can be a part of supporting that culture, and you can leverage it well, by sharing ideas liberally whenever you can.

